# An update on periodontal inflammation and bone loss

**DOI:** 10.3389/fimmu.2024.1385436

**Published:** 2024-06-11

**Authors:** Mingzhu Zhang, Yali Liu, Hamideh Afzali, Dana T. Graves

**Affiliations:** ^1^ Yunnan Key Laboratory of Stomatology, Kunming Medical University, School of Stomatology, Kunming, China; ^2^ Department of Periodontics, School of Dental Medicine, University of Pennsylvania, Philadelphia, PA, United States

**Keywords:** periodontal disease, immune response, microbiota, scRNA-seq, innate immunity, adaptive immunity

## Abstract

Periodontal disease is a chronic inflammatory condition that affects the supporting structures of the teeth, including the periodontal ligament and alveolar bone. Periodontal disease is due to an immune response that stimulates gingivitis and periodontitis, and its systemic consequences. This immune response is triggered by bacteria and may be modulated by environmental conditions such as smoking or systemic disease. Recent advances in single cell RNA-seq (scRNA-seq) and *in vivo* animal studies have provided new insight into the immune response triggered by bacteria that causes periodontitis and gingivitis. Dysbiosis, which constitutes a change in the bacterial composition of the microbiome, is a key factor in the initiation and progression of periodontitis. The host immune response to dysbiosis involves the activation of various cell types, including keratinocytes, stromal cells, neutrophils, monocytes/macrophages, dendritic cells and several lymphocyte subsets, which release pro-inflammatory cytokines and chemokines. Periodontal disease has been implicated in contributing to the pathogenesis of several systemic conditions, including diabetes, rheumatoid arthritis, cardiovascular disease and Alzheimer’s disease. Understanding the complex interplay between the oral microbiome and the host immune response is critical for the development of new therapeutic strategies for the prevention and treatment of periodontitis and its systemic consequences.

## 1 Introduction

Advances in single cell techniques have provided new insight into cell types that are modified in their numbers or activity in subjects with periodontitis. In addition, *in vivo* animal studies have established cause-and-effect relationships through the use of biologic agents or genetically modified mice. Periodontal disease consists of periodontitis and gingivitis, both of which are triggered by bacteria and caused by the host’s immune response. While gingivitis causes inflammation without loss of connective tissue attachment or bone, periodontitis leads to the destruction of the connective tissue attachment and alveolar bone (1–3). Periodontal disease has a significant impact on oral health and has been implicated as contributing to the pathogenesis of several systemic conditions, including diabetes, Alzheimer’s disease, rheumatoid arthritis, and cardiovascular disease (2–5).

Periodontitis involves the activation of the inflammatory response caused by a change in bacteria, generally referred to as dysbiosis (6, 7). The nature of dysbiosis is not well defined and represents one of the major challenges in oral health research. Readers are referred to recent reviews examining microbial dysbiosis that precedes the development of periodontitis (5–8). Key cell types in the initial response to bacteria include keratinocytes and stromal fibroblasts, which are not typical immune cells (9, 10). The interaction of these cells with immune cells leads to gingival inflammation and the initiation of pathways that damage connective tissue. In gingivitis, the loss of connective tissue is reversible. In some individuals, gingivitis leads to periodontitis. The factors responsible for this transition have not yet been well defined although recent results provide new information on potential cell-cell communications that are involved.

Single cell RNA sequencing provides transcript level analysis of cells that have been isolated from tissues. This approach is particularly useful because it provides an unbiased examination of hundreds of transcripts in each cell that gives insight into the cell type, cell state, and cell activity. Taken together, the scRNA-seq data has defined key subpopulations of stromal cells, keratinocytes and leukocytes and their potential mechanistic role in periodontitis. However, it is important to consider several key limitations of this approach. The method used to isolate cells from gingival tissue can lead to selective loss or enrichment of certain cell populations, influencing the results. The depth of sequencing can also bias the identification of highly expressed genes over those with lower transcript levels. Furthermore, the arbitrary determination of cell clusters can result in differences in the number of clusters reported by different investigators. Finally, the high cost of scRNA-seq limits the number of biological replicates that can be examined, necessitating confirmation of findings through alternative approaches. Awareness of these caveats is crucial when interpreting scRNA-seq data in the context of periodontitis research.

## 2 Innate immunity

### 2.1 Epithelial barrier

The function of epithelial tissues is the protection of the organism from chemical, microbial, and physical challenges which is indispensable for viability (10). Keratinocytes form a barrier through tight junctions, adherens junctions, and gap junctions. Bacteria, in turn, can disrupt the epithelial barrier by inducing leukocytes to produce proteolytic enzymes that degrade inter-epithelial junctions, inflammatory cytokines that downregulate the expression of adhesion molecules and keratinocyte apoptosis that disrupts a continuous barrier (10–12). Bacteria can penetrate epithelial cells and reach the basal layer within 24 hours (13). The severity of periodontitis is positively correlated with the extent of epithelial tissue damage. A reduction of epithelial cells is found in moderate or severe periodontitis (14). Bacterial invasion of the oral epithelium causes increased ROS production, which can lead to mitochondrial damage and accumulation and the production of pro-inflammatory factors (15). *P. gingivalis* can modulate gingival keratinocytes to enhance mRNA levels of inflammatory factors and stimulate apoptosis (16) and degrade the proteins that form intercellular adhesions (17, 18). Other invasive bacteria include *A. actinomycetemcomitans*, *T. denticola*, and *F. alocis* (19). The use of protease inhibitors such as leupeptin has been shown to partially mitigate the loss of barrier function induced by *P. gingivalis*, implicating the involvement of microbial proteolytic enzymes in disrupting the epithelial barrier (16).

In addition to providing a physical barrier to microorganisms as part of the host immune defense, the gingival epithelium also expresses a variety of pattern recognition receptors (PRRs) that enable it to recognize microbiota-associated molecular patterns (MAMPs) and respond by secreting cytokines, chemokines and antibacterial peptides. Keratinocytes in the epithelial barrier play a key role in the initiation of the host immune response.

Single-cell RNA sequencing analysis has identified gingival epithelial subpopulations that contribute to inflammatory signatures, antimicrobial defense and neutrophil recruitment in periodontitis (14, 20). scRNA-seq analysis of gingiva indicates an overall reduction in epithelial cells in subjects with periodontitis (14, 20, 21). Caetano et al. identified ten subpopulations of gingival epithelial cells in periodontitis (14). These subpopulations comprised two basal cell clusters, three epithelial clusters expressing high levels of cell cycle genes, one cluster expressing genes associated with extracellular matrix organization and angiogenesis, and four distinct gingival epithelial subpopulations with transcriptomes linked to immune regulation. The latter express transcripts that encode factors that stimulate B-lymphocyte receptor signaling and neutrophil-mediated immunity. Although the overall population of epithelial cells decreased, there was an increase in immune-related epithelial subpopulations (14). Williams et al. identified three keratinocyte subpopulations including a basal cell cluster, a cluster enriched in genes involved in cornification, and a cluster with a gene expression profile consistent with inflammatory responses (20). The gene expression profiles of these cells indicated a shift towards an inflammatory state, with upregulated pathways related to antimicrobial responses and cytokine biosynthesis in subjects with periodontitis (20). Thus, there is an increase in gingival epithelial subpopulations with pro-inflammatory gene signatures with periodontitis and an overall reduction of non-inflammatory epithelial cells. A distinctive junctional epithelial population was characterized by elevated expression levels of serum amyloid A-proteins (SAA) (21). These proteins were found to trigger the secretion of inflammatory cytokines through interaction with the TLR2 pathway in human gingival fibroblasts.

### 2.2 Stromal cells in periodontitis

**Figure 1:**
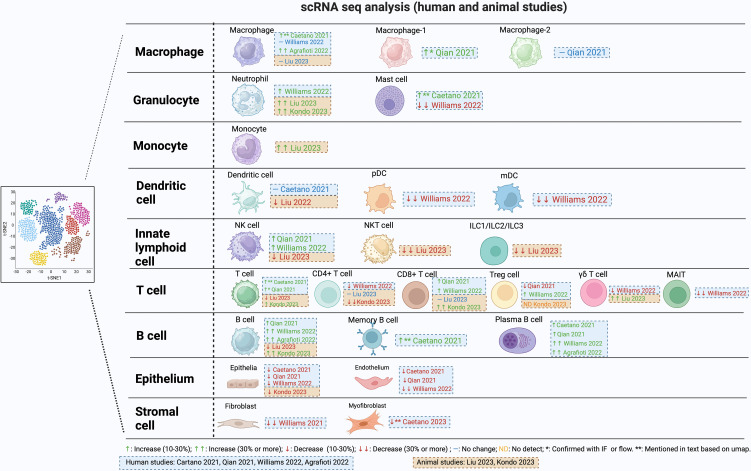
Changes in cell population during periodontitis from single cell RNAseq studies.

Stromal cells in the gingiva consist of mesenchymal stem cells, pericytes and fibroblasts and contribute to tissue integrity, immune regulation, and repair processes. They express several receptors needed to recognize microbes and produce cytokines and chemokines in response. Approximately 30 years ago Yu and Graves suggested that gingival fibroblasts through the production of CCL2 could play an important role in recruiting monocytes and macrophages to inflamed gingiva (22). scRNA-seq data supports the concept that fibroblasts are an important part of the host response. Williams et al. characterized five distinct fibroblast subclusters (20). Among these clusters, two exhibited a transcriptome profile linked to matrix synthesis and tissue remodeling, while the remaining three featured gene signatures associated with immune functions, including leukocyte proliferation, granulocyte migration, and complement activation. Notably, individuals with periodontitis showed a general decrease in fibroblast subpopulations but displayed a specific increase in inflammatory fibroblast subpopulations in parallel with findings with epithelial cells (**Figure 1**). There was an upregulation of genes associated with neutrophil recruitment such as CXCL1 and CXCL8. Caetano et al. identified five distinct fibroblast populations, one pericyte population, and one myofibroblast subpopulation (14). Among the fibroblasts, three subpopulations exhibited enrichment of genes associated with extracellular matrix production, while two other fibroblast subpopulations displayed an inflammatory profile. In the context of periodontitis, a marked reduction was observed in the myofibroblast and pericyte subpopulations, accompanied by an increase in inflammatory fibroblasts, while the other subpopulations remained unchanged. Consequently, both investigations identified a decline in fibroblast numbers, accompanied by an expansion of pro-inflammatory stromal cells in subjects with periodontitis. This data suggests a unique restructuring of the epithelial and stromal compartments in periodontitis, with a specific emphasis on facilitating neutrophil recruitment.

### 2.3 Innate immune cells

#### 2.3.1 Neutrophils

Neutrophils are abundant in gingiva and the gingival sulcus. They have strong antibacterial activity, making them important in the defense against oral infections. Neutrophils may be drawn to the periodontium and other areas that are infected, inflamed, or injured, promoting periodontitis (23). Inflammation increases when neutrophils die and are not quickly removed (23, 24). Neutrophils have regulatory effects on other cell types such as macrophages through production of chemokines that attract macrophages to sites of inflammation and cytokines that modulate polarization of macrophages to an M1 phenotype (25). Upregulation of genes that promote necroptosis, pyroptosis, and ferroptosis-related processes has been reported in neutrophils in subjects with periodontitis (26). When neutrophil recruitment is reduced there is regression of gingival inflammation (27). In contrast, if neutrophil recruitment is totally blocked there is increased severity of periodontitis in humans and in murine periodontitis models, demonstrating the importance of these cells in protecting against oral bacteria. Moreover, the absence of neutrophils causes a secondary increase in IL-17 that leads to greater periodontal inflammation and bone loss (28). The study of neutrophils by scRNA-seq is complicated by their high rates of apoptosis during cell isolation (14). However, a significantly higher proportion of neutrophils was documented in subjects with periodontitis (20) and in periodontitis mouse models (29, 30) (**Figure 1**). It would be helpful if investigators presented differences in actual cell numbers and differences in percentages of cells in supplemental tables, but this has not been done on a consistent basis.

Phagocytosis of pathogens by neutrophils is important in their antibacterial activity (31). Degranulation is one of the ways in which neutrophils exert their anti-microbial and immunomodulatory functions and is important for the progression of periodontitis. The granules are also involved in the inflammatory response and destruction of periodontal tissues through the release of matrix metalloproteinases (MMPs) that break down the extracellular matrix (32) or elastase that disrupts the periodontal epithelial barrier through the cleavage of cell adhesion molecules (33).

Neutrophils produce neutrophil extracellular traps (NETs). Which are a unique DNA structure decorated with antimicrobial peptides (34). Periodontitis is characterized by elevated levels of NETs and delayed NET clearance, compared to healthy gingiva (35). They are produced in response to pathogens and are thought to protect the host by trapping microorganisms, restricting their spread from initial sites of infection, or neutralizing virulence factors (36, 37). Mice that cannot produce NETs are more susceptible to infection (38).

Mast Cells

Mast cells (MCs) are a related granulocyte that can also affect periodontitis negatively (39, 40). Mast cells play a pivotal role in inflammatory responses and can induce bone resorption. They release proteases and histamine from the cytoplasmic granules, as well as cytokines and chemokines. Mast cell counts increase in subjects with chronic periodontitis and in animal studies, mast-cell-deficient mice have significantly reduced alveolar bone loss, demonstrating a cause-and-effect relationship (40, 41). In human gingival tissue, one scRNA-seq publication reported an enrichment of mast cells in subjects with periodontitis compared to non-periodontitis (14), while another report noted a decrease (20) (**Figure 1**). The basis for this difference is unknown.

#### 2.3.2 Monocytes/Macrophages

Monocytes and macrophages are important in periodontal destruction. Monocytes from the blood reach the tissue microenvironment and develop into macrophages (42). The phenotypic transformation of macrophages plays an important role in the immune response during the onset, development, and regression of periodontitis (43, 44). Macrophages can be polarized to at least two different types with opposite activities: M1-type macrophages and M2-type macrophages. M1 macrophages produce high levels of pro-inflammatory cytokines, and may exacerbate inflammation and tissue damage. M2 macrophages produce anti-inflammatory molecules and growth factors. They participate in processes such as tissue repair, regeneration, and inflammation resolution (43, 44). The onset of periodontitis is linked to the formation of M1 macrophages that are pro-inflammatory and can promote osteoclast differentiation by stimulating the production of RANKL. When M1 polarization is blocked, there is reduced periodontal bone loss (45, 46). M2 macrophages exert anti-inflammatory effects in periodontal tissue by producing IL-10 and TGF-b (47, 48). Controlled release of particles that contain CCL2 induce polarization of M2 macrophages reduce RANKL expression and osteoclast numbers, thereby inhibiting alveolar bone loss (49). Single-cell RNA sequencing data in periodontitis reveals a significant increase in macrophages compared to healthy individuals (14, 50), (**Figure 1**). Interestingly, macrophages in periodontitis express both pro-inflammatory and anti-inflammatory markers, challenging the notion of exclusive polarization (50).

#### 2.3.3 Dendritic cells (DCs)

DCs connect innate and adaptive immunity by capturing antigens and inducing antigen-specific immune reactions (51). There are two major classes of DC, monocytoid (mDC) that are of monocyte lineage and plasmacytoid (pDC) which are of lymphocytic origin (52). mDC are also known as conventional DC (cDC). cDC are primarily activated in response to bacterial infection and pDC in response to viral infection (51). Human studies show that the number of cDCs decline and pDCs rise in the gingiva of subjects with periodontitis (53). DCs are crucial in guiding naïve T-cells towards T helper cell (Th1, Th2, Th17, Treg, Tfh) differentiation (54) and activating CD4 and CD8 immune responses. They also up-regulate activity in monocytes/macrophages, neutrophils, and NK cells (51, 52).

A reduction in DC function increases susceptibility to periodontitis (55). Without adequate DC activity, the production of antibodies in response to bacterial challenge is significantly reduced (55, 56). Reduced activity of DCs results in a compensatory increase in the expression of inflammatory and pro-osteoclastogenic factors, IL-1β, IL-17, and RANKL (56). Conflicting results have been obtained in scRNA-seq analysis of DC. One publication found there was no clear difference in this cell type between healthy and periodontitis subjects (14) while another (20) reported a notable reduction of ~ 30% in both pDC and cDC in individuals with periodontitis (**Figure 1**). The reason for this difference is unknown but could involve differences in cell isolation and the parameters of cell clustering.

Langerhans cells (LCs) are a subset of dendritic cells found in the epithelium of mucosa and skin tissues. They respond to both mechanical and bacterial stimulation and play a role in the development of mucosal immunity (57). Depletion of LCs accelerates periodontal bone loss (57, 58) agreeing with increased susceptibility when DC function is compromised (55). Smoking has been found to specifically diminish gingival LCs in healthy individuals, raising the possibility that the loss of LCs may contribute to periodontitis in smokers (53).

## 3 Innate lymphoid cells

### 3.1 NK T-cells, γδ T-cells and MAIT cells

Unconventional T-cells include natural killer T (NKT) cells, γδ T-cells and mucosal-associated invariant T (MAIT) cells that express CD3. They are more prevalent in gingiva from subjects with periodontitis (59). NK T-cells are a specialized subset of T-cells with αβ T-cell receptors (TCRs) and NK cell receptors on their surface (59, 60). Gram-negative bacteria possess glycosphingolipids that can activate NK T-cells via antigen presenting cells. NK T-cells can enhance RANKL production, osteoclastogenesis, and alveolar bone loss in mice following oral *P. gingivalis* inoculation and in other models (61, 62).

γδ T-cells express T-cell receptors (TCR) consisting of the gamma and delta chains, with a limited diversity, and do not express CD4 or CD8 (63). These cells are stimulated by a variety of signals, such as direct antigen binding to TCR, stimulation of toll-like receptors or cytokine stimulation. They are found in the epithelium or in the connective tissue adjacent to the epithelium and make up the majority of T-cells in epithelial tissues (64). γδ T-cells are elevated in inflamed human gingiva (64), and are increased to a greater extent than αβ T-cells (64). γδ T-cells stimulate the recruitment of macrophages and neutrophils and produce IL-17A and IFNγ (65). In mice, they are the principal source of IL17A. In the oral inoculation model of periodontitis, γδ T-cells have distinct pathogenic functions, and their reduction significantly reduces loss of alveolar bone. However, this linkage does not exist in the ligature model, pointing out an important difference in the two primary murine models of periodontitis (65). scRNA-seq data reveals an approximately 20% decrease in γδ T-cells among all cell types in individuals with periodontitis compared to healthy individuals (20), contrasting with a 30% increase among immune cells observed in a mouse model of periodontitis compared to the healthy state (29) (**Figure 1**). Using different reference populations could be a potential reason for the contrasting findings and species differences (64). Another difference may be due to the fact that most human studies represent inflamed tissue that may not exhibit current disease activity, a significant limitation in most human studies, whereas the disease activity is typically progressing in murine models (66).

MAIT have a restricted T-cell receptor (TCR) response (67). Notably, TCR-independent mechanisms such IL-18 signaling can activate MAIT cells. They differ in how they react to various microorganisms, and this diversity may help them discriminate between dangerous pathogens and beneficial commensal species. Germ-free mice have fewer MAIT cells and MAIT cell populations increase during infection, suggesting a protective function against microbial challenge (67). Emerging evidence indicates that MAIT cells may contribute to the development of periodontitis by producing proinflammatory cytokines like IL-17 and TNF when activated by pathogenic microorganisms in the oral cavity. Further research is required to comprehensively elucidate the precise role of MAIT cells in periodontitis (68). A scRNA-seq study reported a significant decline in MAIT cells in subjects with periodontitis (20) The scRNAseq data revealed an upregulation of nucleotide oligomerization domain (NOD)−like receptor signaling pathways, apoptosis, IL-17 signaling, and TNF signaling in MAIT cells from periodontitis subjects (69).

### 3.2 Innate lymphoid cells-1, -2 and -3.

Lymphoid cells that lack T-cell receptors but are of lymphocyte lineage include innate lymphoid cells (ILC) with three subtypes (ILC1, ILC2, and ILC3). Rather than reacting to antigen, these cells react directly to signals of stress and danger. They possess pattern recognition receptors (TLR2, TLR4, TLR9, NLRP3, RAGE, P2X7 and P2Y2, etc) on their cell surface that respond to danger-associated molecular patterns (LPS, S100 proteins, AGEs, ATP, ROS, etc). ILCs are primarily tissue-resident cells and are classified according to the cytokines they generate (70). ILC1 cells express similar cytokines to Th1 cells such as IFNγ, ILC2 cells produce cytokines similar to Th2 cells such as IL-4, IL-5, IL-9, and IL-13 and ILC3 cells produce IL17A, similar to Th17 cells. According to scRNA-seq data, a mouse model of periodontitis exhibits a reduction of over 30% in ILC cells among immune cells in animals with induced periodontitis (29). ILC1 was the predominant subset, comprising over 60% of ILCs in mice and humans. In humans, the percent ILCs were not significantly altered in the gingiva of subjects with periodontitis. Notably, a small proportion of ILC1 cells expressed RANKL and and ILC3 produced IL17A suggesting they could participate in bone resorption (71). The plasticity, differentiation, tissue-specific migration and accumulation of ILC subpopulations may be an important modulator of the local immune response (72).

### 3.3 Natural killer (NK) cells

NK cells are lymphocytes belonging to the innate immune system (60). NK cells are cytolytic, killing viral or bacterial-infected, or malignant cells, and can exert pro-inflammatory effects. Through the release of granzymes and perforin, NK cells directly destroy their targets. NK cells produce cytokines like IFN, TNF, IL-5, IL-13, and GM-CSF that upregulate activity in other cells, particularly macrophages and contribute to the control of infections (60, 73) (**Figure 1**). NK cells have a role in senescent cell clearance. They are stimulated by bacteria through toll-like receptors (TLRs) and cytokines produced by cells such as dendritic cells (73).

NK cells tend to have proinflammatory influences in periodontitis (60). This is manifested through cytokine production, cytotoxic effects, and dendritic-cell crosstalk. Moreover, increased numbers of NK cells in patients wtih periodontitis and decreased numbers after periodontal therapy have been observed (60). Additionally, NK cells are correlated to the regulation of T-cell proliferation and suppression of B-cells in periodontitis (60). Overall, these findings suggest that NK cells play a role in the pathogenesis of periodontitis, particularly through their proinflammatory influences (60).

## 4 Adaptive immunity

### 4.1 CD4+ T-cells

Naive CD4+ T-cells are capable of differentiating into a variety of functional and phenotypical T helper (Th) cell subsets, Th1, Th2, Th17, Treg and Tfh cells (67, 74). Th1 cells are pro-inflammatory and produce IL-1β and IFN-γ to promote inflammation and are associated with tissue damage in periodontitis. Th2 cells play a key role in the production of antibodies. Interleukin-4 (IL-4) and other Th2-cell-derived cytokines are anti-inflammatory and are considered to reduce bone loss. However, antibodies produced by Th2 responses activate complement and could potentially be pro-inflammatory. Th17 cells promote inflammation through IL-17 production. Th17 cells are increased in human periodontitis; reducing Th17 cell numbers reduces alveolar bone resorption in experimental periodontitis (75). In humans, Th17 cells are the principal source of IL17A. IL-17 stimulates osteoblast-lineage cells to secrete RANKL and GM-CSF to enhance osteoclast formation and bone resorption (76). IL-17A also stimulates fibroblasts, epithelial cells, and endothelial cells to produce RANKL, MMP, PGE2, and chemokines to promote the progression of periodontitis (77). IL-17A can affect immune cells such as macrophages, neutrophils, dendritic cells, and B-cells. Regulatory T-cells (Treg) stimulate immunosuppression and resolution of inflammation through production of TGF-b, cytotoxic T lymphocyte-associated protein 4 (CTLA-4), lymphocyte activation gene 3 (LAG-3), programmed cell death protein 1 (PD1), T-cell immune receptors with Ig and ITIM structural domains (TIGIT), and T-cell immunoglobulin and mucin-containing structural domain 3 (Tim-3) (78). The percentage of these cells increases in the later stages of periodontitis to reduce disease progression and reestablish homeostasis (79). Interestingly, increased RANKL promotes the induction of Tregs and increases formation of M2 macrophages, thus facilitating the resolution of inflammation (79). T follicular helper (Tfh) cells play an important role in the regulation of humoral immunity and germinal center responses, and in periodontitis, may promote local B-cell activation, and maintain a long-term humoral immune response (80). Tfh cells in older individuals may contribute to increased inflammation in periodontitis (74).

Th22 cells are a subpopulation of T-helper cells that produce IL-22 and TNF, which have been linked to the pathogenesis of periodontitis by increasing inflammation (81) and the number of Th17 cells in periodontal lesions (82). Oral inoculation of bacteria in mice stimulates the production of IL-22 through increased numbers of IL-22-expressing CD4+ T-cells in periodontitis-affected tissues (83). This increase is associated with higher levels of RANKL and alveolar bone resorption.

scRNA-seq analysis indicates that T-cells constitute the largest lymphocyte population, followed by B-cells and plasma cells (20) (**Figure 1**). In human samples with periodontitis there is an overall expansion of T-cells (20, 21). Various studies have identified distinct subclusters of T-cells within single-cell RNA sequencing datasets from both healthy and diseased conditions. These subclusters include CD4+, MAIT, CD8+, γδ T-cells, Treg, TH17, and NK T-cells, which are consistently observed across different studies, albeit with varying proportions in periodontitis (20, 21, 29). Human studies indicate an approximately 25% decrease in CD4^+^ T-cells in human subjects (20) and a similar reduction of over 30% in mouse periodontitis models (30).

### 4.2 CD8+ T lymphocytes

CD8+ T-cells kill virally or bacterially infected cells. CD8+ T-cells are fewer in number than CD4+ T-cells in periodontitis lesions (84). CD8+ cytotoxic T lymphocytes produce TNF, IFN-γ and kill cells through expression of Fas ligands, pore-forming proteins (perforins) and proteases (granzyme) (85). CD8+ regulatory T lymphocytes (CD8+ Tregs) produce CTLA4, TGF-b and IL-10 to resolve inflammation. Systemic administration of CTLA-4 reduces alveolar bone resorption in experimental periodontitis (86). Like pro-inflammatory CD4+ T-cells, the pro-inflammatory CD8+ cytotoxic T lymphocytes likely promote periodontitis whereas the pro-resolving CD8+ Tregs help prevent or reduce it. In human gingival tissue, scRNA-seq studies indicate a small ~10% increase in CD8+ T-cells in subjects with periodontitis compared to non-periodontitis subjects (20, 21). Interestingly, periodontitis was associated with an increase in expression of CCL4, CCL4L2, and CCL3L3 in both CD8 T-cells and NK cells. Elevated levels of the CCR5 ligands in cytotoxic CD8+ T-cells underscores their potential role in recruiting inflammatory cells during periodontitis (21).

### 4.3 B lymphocytes

B-cells are part of the humoral component of the adaptive immune system and are specialized in producing antibodies. B-cells can also present antigens and enhance inflammation through cytokine production, opsonization, and complement fixation mediated by the antibodies they produce. B-cells and plasma cells are increased in periodontitis (87). Hub genes are located at critical nodes in biological processes such as chronic inflammation. Interestingly, a recent study pointed to B-cells as expressing a high number of hub genes that are correlated with inflammation in periodontitis (88). B-cells produce RANKL to promote bone loss (79). Evidence that B-cells contribute to periodontitis was shown in a ligature-induced murine model in which there was significantly less bone loss in B-cell deficient mice (89). On the other hand, B-cells can potentially reduce periodontitis by limiting bacterial invasion. In support of the latter, reduced dendritic cell activation of B-cells increases periodontitis in an oral inoculation murine model (55). B regulatory cells (Bregs) can reduce inflammation and limit excessive inflammatory responses similar to Tregs. Bregs produce anti-inflammatory cytokines such as IL-10, and inhibit alveolar bone resorption (90, 91). Plasma cells that produce anti-inflammatory cytokines IL-35 and IL-37 also inhibit alveolar bone loss (92). Taken together, evidences suggests that B lymphocytes have a dual role in modulating the progression of periodontitis and can both promote and inhibit alveolar bone resorption depending on the specific conditions.

scRNA-seq studies observed an overall increase in the proportion of B-cells in human subjects with periodontitis compared to healthy controls (21, 50). Three distinct B-cell populations were consistently detected including memory B-cells, IgG-producing plasma B-cells, and follicular B-cells (20). Caetano et al. (14) reported a distinct increase in memory B-cells in moderate periodontitis compared to healthy individuals. The increase is backed up by several publications using alternative approaches showing there is a significant increase in plasma cells in periodontitis compared to healthy individuals (14, 20, 21, 50) (**Figure 1**).

## 5 Osteoblast lineage cells and periodontitis

In a periodontally healthy adult, an episode of bone resorption is followed by an equivalent amount of bone formation, which is referred to as coupling. In periodontitis, chronic inflammation inhibits bone coupling after an episode of bone resorption, increasing the size of an osteolytic lesion (2, 93). Thus, osteolytic lesions occur due to bone resorption and inhibition of coupled bone formation. Immune activation significantly reduces coupled bone formation (93). Experimental animal models have demonstrated that oral microbial dysbiosis stimulates inflammation by in bone-lining cells and osteocytes by enhancing nuclear translocation of NF-κB (2). Lineage-specific inhibition of NF-κB in osteoblasts and osteocytes, but not in other cell types, mitigates periodontal bone loss caused by dysbiosis (94). This phenomenon can be attributed to two primary mechanisms. Firstly, inhibition of NF-κB activation reduces RANKL expression in osteocytes and osteoblastic cells, resulting in reduced bone resorption. Osteocyte production of RANKL is significant due to their location within bone. Secondly, the activation of NF-κB in cells of the osteoblast lineage blocks coupled bone formation. The reduction in coupled bone formation is due to NF-κB’s role in limiting osteoblast differentiation, indirectly inducing apoptosis in osteoblastic cells and through reducing the synthesis of bone osteoid (94, 95). Such increased apoptosis is significant, as treatments targeting apoptosis have been shown to reduce periodontal bone loss by promoting increased coupled bone formation (96). Inflammation also inhibits mesenchymal stem cell (MSC) differentiation into osteoblasts by blocking the upregulation of transcription factors, runt-related transcription factor 2 (Runx2) and osterix (Osx) (3). Therefore, the activation of NF-κB in osteoblast precursors, osteoblasts, and osteocytes play an essential role in periodontitis, contributing to enhanced bone resorption and limiting the process of coupled bone formation.

## 6 Periodontitis and systemic diseases

There is a relationship between periodontitis and systemic conditions such psoriasis, rheumatoid arthritis, inflammatory bowel disease, type-2 diabetes, osteoporosis, non-alcoholic fatty liver disease, Alzheimer’s disease, pre-term birth, cancer progression and cardiovascular disease (97). In some cases there may be an association through co-morbidities and in others a causal relationship. The relationship is often two-way. Periodontal disease may worsen glycemic control and diabetes may enhance periodontal disease progression (4, 5). The latter may be due to bacteremia entering the bloodstream through invasion of the oral epithelial barrier, which may impact systemic disease a distant sites including an effect on hematopoiesis (4, 5, 98).

Periodontal inflammation and bone loss is enhanced by diabetes (99). The diabetic condition promotes the inflammatory response to bacteria (100) and alters the microbial composition to render it more pathogenic (98, 101). Clinical evidence shows that effective periodontal treatment improves blood glucose levels in patients with type 2 diabetes, suggesting a causal relationship between periodontal disease and glycemic control (102, 103). Similar to the link with diabetes, the intersection between rheumatoid arthritis and periodontitis is thought to be bidirectional. Rheumatoid arthritis subjects have greater loss of attachment, and increased expression of inflammatory mediators (IL-17, IL-2, TNF, and IFN-γ) that is linked to an increase in bacterial load and an increase in periodontal pathogens (104). Periodontal disease may contribute to the etiology of rheumatoid arthritis by increasing exposure of subjects to enzymes that citrullinate proteins to stimulate an auto-immune response (105, 106). In addition to the effect of systemic disease on periodontal tissues, bacteremia caused by periodontal disease may cause epigenetic changes in the bone marrow that affect hematopoiesis. Maladaptive bone marrow (BM)-mediated trained innate immunity (TII) has been proposed as a co-morbidity between periodontitis and arthritis (107, 108). In this scenario, chronic inflammation causes epigenetic changes in the bone marrow to increase the inflammatory response at a distant site.

## 7 Summary

Periodontal disease is one of the most common causes of oral inflammation and periodontitis is one of the most common osteolytic diseases found in adults (2). They are triggered by bacteria, although the sequelae are due to the impact of bacteria-induced innate and acquired immune responses. New approaches such as scRNA-seq have provided a new understanding of how immune and non-immune cells have bi-directional communication to initiate and amplify the inflammatory response triggered by bacteria. For example, there are distinct epithelial cell subpopulations that contribute to antimicrobial defense and are likely to play an important role in neutrophil recruitment (14, 20). Subjects with periodontal inflammation experience an overall loss of epithelial cells, but there is an increase in epithelial cells with an inflammatory signature that stimulates neutrophil recruitment. Stromal cells also consist of subtypes that have inflammatory signatures, such as a fibroblast subtype that produces chemokines to stimulate leukocyte recruitment (20, 22). Subjects with periodontitis have a shift towards more pro-inflammatory fibroblast phenotypes, accompanied by a decline in matrix-producing subsets.

Examination of the innate immune landscape reveals complex changes in myeloid cell populations, including neutrophils, macrophages, and dendritic cells. These cells exhibit a balance of protective and destructive functions, depending on their precise activation state. Neutrophils play a crucial role in the pathogenesis of periodontitis, and can be both protective and destructive functions depending on their numbers and activation state (23, 24). While the presence of neutrophils is essential for combating bacterial infection (109) and initiating the inflammatory response, an excessive or dysregulated neutrophil response can contribute to tissue damage and disease progression (26, 27). A substantial reduction in neutrophils, as observed in certain genetic disorders like leukocyte adhesion deficiency, leads to an increased severity of periodontitis (25). Conversely, a large, persistent neutrophil infiltration in periodontal tissues can also exacerbate inflammation and connective tissue breakdown (20). Neutrophils release proteolytic enzymes, such as matrix metalloproteinases (MMPs) and elastase, which degrade the extracellular matrix and disrupt the epithelial barrier. Additionally, the formation of neutrophil extracellular traps (NETs), while initially beneficial for trapping pathogens, can cause collateral tissue damage if not properly regulated and cleared. Therefore, a balanced neutrophil response is critical for maintaining periodontal health, as both the absence and the excessive or chronic presence of these cells can contribute to the initiation and progression of periodontitis.

Macrophages can polarize into distinct phenotypes (43, 44). M1 macrophages promote inflammation, tissue destruction and bone resorption by producing pro-inflammatory cytokines. The onset and progression of periodontitis is closely linked to the formation of M1 macrophages. Conversely, M2 macrophages exhibit an anti-inflammatory phenotype and participate in resolving inflammation and tissue repair by releasing anti-inflammatory cytokines. Single-cell RNA sequencing data from periodontitis lesions reveal a significant increase in macrophages expressing both pro-inflammatory (M1) and anti-inflammatory (M2) markers (45–48). M1 macrophages may drive inflammation and bone loss in the early stages, while M2 macrophages may play a protective role in later stages by resolving inflammation and promoting tissue regeneration.

A number of cell types, particularly lymphocytes with innate immune properties, have recently been identified as contributing to periodontal inflammation and bone loss, particularly. NKT-cells, γδ T-cells, and mucosal-associated invariant T (MAIT) cells (59). In periodontitis, NKT-cells may enhance inflammation, RANKL production, osteoclastogenesis, and alveolar bone loss. γδ T-cells express a distinct TCR composed of gamma and delta chains and play critical roles in barrier surveillance (63, 64). In periodontitis, γδ T-cells are elevated and stimulate the recruitment of macrophages and neutrophils, as well as the production of pro-inflammatory cytokines like IL-17A and IFN-γ. Their absence has been shown to significantly reduce alveolar bone loss in animal models of periodontitis. MAIT-cells are known to increase during infections, suggesting a protective role. Their numbers are reduced in periodontitis, suggesting the loss of a key protective cell type (20, 69). However, this has not yet been functionally demonstrated. Conventional lymphocytes expressing αβ receptors have also been implicated in periodontitis. Anti-inflammatory T-cells (T-regs and B-regs) limit periodontal inflammation and bone loss, while Th1 and Th17 T-cells have been implicated in stimulating inflammation and periodontal bone loss (73, 74, 77). The role of Th2 cells is more complicated as the production of antibodies may be protective or by activating complement, may lead to inflammation-induced tissue damage.

Periodontal ligament fibroblasts and osteoblast lineage cells consisting of osteoblasts and osteocytes are strongly affected by inflammation that leads to periodontitis. One hypothesis of periodontitis links the proximity of periodontal inflammation to bone as a key event that distinguishes gingivitis from periodontitis (92, 93). The onset of periodontitis activates NF-κB signaling, which induces the expression of RANKL in osteocytes, bone lining cells and PDL fibroblasts (110). Interestingly, deletion of RANKL in osteocytes has a dramatic effect on reducing bone resorption stimulated by oral inoculation of P. gingivalis and F. nucleatum. In addition to stimulating bone resorption, inflammation suppresses expression of bone matrix proteins and causes osteoblast cell death leading to disruption of bone coupling (95). Collectively, these inflammatory processes affect osteocytes, osteoblasts, and PDL fibroblasts to induce periodontitis by simulating osteoclast formation and activity and inhibiting repair of osteolytic lesions.

The research presented highlights several important implications for clinical practice in managing periodontitis. The findings related to the involvement of specific cell types and their states at different disease stages could guide the development of stage-specific targeted therapies. Modulating the balance of macrophage polarization towards an anti-inflammatory M2 phenotype, or regulating the recruitment and activation of neutrophils, may help resolve the destructive inflammatory response. The scRNA-seq data could identify valuable biomarkers for monitoring disease progression, predicting treatment responses, and guiding personalized management of periodontitis. Additionally, the insights into cell populations involved in tissue repair and the adaptive immune response could pave the way for developing regenerative therapies. In summary, the insights gained from research highlight the importance of targeting specific cellular and molecular mechanisms involved in periodontitis to develop more effective prevention and treatment strategies, with the potential to also impact various systemic diseases associated with chronic inflammation.

## Author contributions

MZ: Writing – original draft. YL: Writing – original draft. HA: Writing – original draft. DG: Writing – original draft, Writing – review & editing.
